# Analysis of microRNAs and the microRNA-messengerRNA regulatory network in chronic alcohol exposure

**DOI:** 10.3389/fphar.2024.1377501

**Published:** 2024-08-21

**Authors:** Ailin Du, Yingying Chen, Siyu Qiao, Jiaxing Dong, Yulin Li, Bokai Cao, Rongyu Zhao, Ruiling Zhang

**Affiliations:** ^1^ Sino-UK Joint Laboratory of Brain Function and Injury of Henan Province, Henan International Joint Laboratory of Noninvasive Neuromodulation, Department of Physiology and Pathophysiology, School of Basic Medical Sciences, Xinxiang Medical University, Xinxiang, China; ^2^ Department of Psychiatry, The Second Affiliated Hospital of Xinxiang Medical University, Xinxiang, China; ^3^ Department of Gastrointestinal Surgery, Shanghai East Hospital, School of Medicine, Tongji University, Shanghai, China

**Keywords:** chronic alcohol exposure, differential expression, regulatory network analysis, miRNAs, mRNAs, gene expression, bioinformatic analysis, gene enrichment

## Abstract

**Introduction:** Chronic alcoholism is one of the most common neurological diseases in modern society. However, the key mechanisms underlying learning and memory impairments caused by chronic alcohol exposure remain unclear. In this study, a microRNA-messenger RNA (miRNA-mRNA) network was constructed to explore the potential function of key genes in chronic alcohol exposure, their effects on the hippocampus, and their mechanisms which facilitate brain injury in mice.

**Methods:** The Morris water maze test was used to assess the learning ability of mice in each group. Mitochondrial ATPase activity and H_2_S levels in the hippocampi of mice were determined. Differentially expressed miRNAs and mRNAs in the mouse hippocampus were identified using second-generation sequencing. Using the TargetScan, miRTarBase, and miRDB databases, we predicted miRNA target genes and constructed a miRNA-mRNA regulatory network. Furthermore, using the Gene Ontology and KEGG databases we performed functional enrichment and protein-protein interaction analyses. Real-time quantitative polymerase chain reaction (qPCR) and other methods were employed to verify the mRNA expression of related genes.

**Results:** The Morris water maze test revealed that mice exposed to chronic alcohol exhibited a significantly reduced learning ability compared to the control group (*p* < 0.05). Compared with the control group, the activity of mitochondrial ATPase in the hippocampal tissue of alcohol-treated mice was significantly decreased (*p* < 0.01), suggesting brain injury. In the model group, H_2_S significantly increased in the mice hippocampi (*p* < 0.01), indicating that chronic alcohol exposure could activate cystathionineβ-synthase (CBS) and catalyze the mass formation of H_2_S, suggesting brain injury. A total of 208 differentially expressed miRNAs and 377 differentially expressed mRNAs were screened through bioinformatic analysis. Enrichment analysis indicated that the main pathways were involved in neurodegeneration and regulation of the Wnt signaling pathway. The PCR detected a significant downregulation in the expressions of *FOS* and *EGR1* genes.

**Discussion:** Consequently, chronic alcohol exposure may regulate the expression of *FOS* and *EGR1* in the hippocampus through *miR-222-3p, miR-132-3p, miR-212-3p,* and *miR-191-5p*, reduce the activity of hippocampal mitochondrial ATPase, activate CBS, catalyze the large amount of H_2_S formation, and destroy the mitochondrial structure, resulting in decreased learning ability. Our findings revealed valuable genes and miRNAs for the study of chronic alcohol exposure.

## 1 Introduction

In modern society, chronic alcoholism is a major threat to health. The accepted definition of chronic alcohol exposure is excessive and persistent alcohol consumption that affects one’s health, relationships, legal status, or work (Ormerod et al., 1988). Alcohol kills >3.3 million individuals globally every year (Pervin and Stephen, 2021), accounting for 3.8% of the global annual mortality rate (Marroni et al., 2018). Chronic alcoholism can lead to the weakening or loss of social willpower, hypnosis, lethargy, coma, and other disturbances of consciousness. It is complicated by atherosclerosis and other diseases, and may even result in death due to respiratory and blood pressure suppression (Fuchs and Fuchs, 2021). Studies have shown that long-term heavy drinking causes varying degrees of nervous system damage, resulting in cognitive impairment and brain damage (Mukherjee, 2013). Its influence on the central nervous system (CNS) manifests in motor coordination disorders, sleep induction, anxiety, forgetfulness, and other ailments (Mukherjee, 2013).

MicroRNAs (miRNAs) are small (19–23 nucleotides) non-coding RNAs. As post-transcriptional regulators of gene expression, miRNAs are widely regarded as universal regulators of functional characteristics of various cancers, including proliferation, apoptosis, invasion, metastasis, and genomic instability (Chen et al., 2022). Recently, an increasing number of studies have indicated that miRNAs play an important role in learning. Inhibition of *miR-124* expression can restore the miR-124/PTPN1 pathway, thereby restoring synaptic failure (Hou et al., 2020). Therefore, miRNAs are likely involved in the mechanism of learning impairment caused by chronic alcohol exposure.

In recent years, pathological studies on neurodegenerative diseases of the brain caused by chronic alcoholism have laid the foundation for bioinformatics studies on differentially expressed genes following chronic alcohol exposure (Coleman et al., 2017). Recent studies have shown that *miR-3473b* and *miR-1944-3p* are upregulated in thrombin-stimulated olfactory mucosal mesenchymal stem cells, and are associated with alcohol exposure (Gao et al., 2023). This study aimed to investigate the potential mechanism underlying chronic alcohol exposure by measuring ATPase activity and H_2_S content in the hippocampal mitochondria of mice chronically exposed to alcohol. In this study, a miRNA-mRNA regulatory network related to chronic alcohol exposure was constructed, and a functional enrichment analysis was performed to explore the role of miRNAs in the mechanism of hippocampal learning injury in mice chronically exposed to alcohol. Key genes were verified using quantitative polymerase chain reaction (qPCR) to provide valuable information for further elucidation of learning impairments caused by chronic alcohol exposure.

## 2 Materials and methods

### 2.1 Experimental animals

Twenty male C57BL/6 mice with no significant difference in learning ability were screened using the water maze pre-test, and divided into control and model groups, with 10 mice in each group, according to the random number table method. We used ≥99.5% anhydrous ethanol (Aladdin, China) and distilled water to get a 10% (V/V) alcohol solution. The model group had free access to 10% (V/V) alcohol solution. After continuous access to 10% alcohol solution for 60 days, in accordance with previous studies, the water maze test and other experiments were used to verify the learning and memory impairments and determine whether the mouse model of chronic alcohol exposure was established (Salling et al., 2016). The control group had free access to the same volume of distilled water as the alcohol solution. The two groups of mice were bred in the same environment and conditions, with free drinking intake, but were separated into different cages with general access to 10% alcohol solution. Animal experiments in this study were approved by the Animal Ethics Committee of the Xinxiang Medical University, and all experiments strictly followed the 3R principles.

### 2.2 Main instruments

The main instruments used in this study include a qTOWER2.2 fluorescent qPCR instrument (Analytik Jena, Germany), centrifuge D3024R (Scilogex, United States), ordinary PCR instrument EasyCycler (Analytik Jena, Germany), UV-visible spectrophotometer UV-1800 DS2 (Mapada, China), ScanDrop100 (Analytik Jena, Germany), high-speed refrigerated centrifuge (Heraeus, Germany), and ELx800 Automatic Microplate Tester (BioTek, United States of America).

### 2.3 Main reagents and supplies

TruScript 1st Strand cDNA Synthesis kit (Ai Delai); Ultrafine total ATPase determination, protein quantitative test, mitochondrial extraction (Nanjing Jiancheng Institute of Biological Engineering, China) kits; 2 × SYBR^®^ Green premix (China DF); 10 μL tip (GCS, United States of America), 200 μL tip (GCS, United States of America), 1 mL tip (United States of America, GCS), 1.5 mL RNA-free EP tube (GCS, United States of America), low white PCR reaction tube (Bio-RAD, United States of America), optical sealing film (Bio-RAD, United States of America) (The gun head and EP tube were sterilized and dried before use), and ethanol (Aladdin, China).

### 2.4 Place navigation experiment

The Morris water maze experiment: experimental animals were allowed to swim and learn to find a platform hidden in the water (Tian et al., 2019). A place navigation experiment was used to measure the learning ability of mice in the water maze. The experiment lasted for 4 days. The route map and time required for the mice to find and climb the platform were observed and recorded, and the incubation period and swimming speed were recorded. The mice were placed in the water with their heads facing the pool wall. One of the four starting positions (East, West, South, and North) was randomly selected, and the time (s) when the mice found the underwater platform was recorded. In the previous training session, if the time exceeds 60 s, the mice were guided to the platform. The mice were allowed to remain on the platform for 10 s, and the incubation period was recorded at 60 s. After each training session, the mice were dried and placed under a 150 W incandescent lamp for 5 min when necessary, and then returned to the cage. Each mouse was trained four times a day with an interval of 15–20 min between two training sessions for four consecutive days. The incubation period was then calculated for each group.

### 2.5 Determination of mitochondrial ATPase activity in the hippocampus using spectrophotometry

All mice were euthanized and the part of the hippocampus were taken from eight mice in each group. Mitochondria were isolated using a mechanical method involving low-speed differential centrifugation to remove debris and large organelles, followed by high-speed differential centrifugation according to the instructions of the mitochondrial extraction kit. After the protein concentration of the sample was measured according to the instructions of the protein quantitative test kit, the samples were successively pre-treated, and enzymatic and phosphorus-fixing reactions were performed, according to the instructions of the ultrafine total ATPase assay kit. Absorbance was determined at a wavelength of 636 nm with a spectrophotometer, according to formula U = [∆A/(0.01 × t)] × D (where U is the enzyme activity, △A is the change of absorbance value during the reaction time, t is the reaction time [min], and D is the dilution multiple) to calculate the ATPase activity.

### 2.6 Indirect determination of H_2_S in the hippocampus using spectrophotometry

Samples of the part of hippocampus were taken from eight mice in each group. The blood on the surface of hippocampus tissue was washed with 50 mmol/L of potassium phosphate buffer pre-cooled by ice, then dried with filter paper, weighed, and stored −70°C. A homogenate solution (mass volume ratio: 0.12) was prepared with 50 mmol/L potassium phosphate buffer (pH 8.0) at 0°C–4°C. The homogenate solution was centrifuged at 12,000 rpm at 4°C for 10 min. The supernatant (75 μL) was placed into another centrifuge tube. Approximately 0.25 mL of zinc acetate solution was added to volume fractions of 0.01 and 0.45 mL distilled water at room temperature and incubated for 10 min. Approximately 0.25 mL trichloroacetic acid was added to a volume fraction of 0.1 mL of distilled water and centrifuged for 10 min at 7,000 r at 4°C. The supernatant was collected and 133 μL of N, N-dimethyl-p-phenylenediamine hydrochloride (20 mmol/L)-hydrochloric acid (7.2 mol/L) buffer and 133 μL of ferric chloride (30 mmol/L)-hydrochloric acid (1.2 mol/L) buffer solution were added fully mixed. After 20 min, absorbance was measured at 670 nm wavelength using an automatic microplate reader. Brain tissue H_2_S was calculated according to the H_2_S standard curve, and the level was expressed as the amount of H_2_S per unit mass of tissue (nmol/g).

### 2.7 High throughput miRNA and mRNA sequencing

Six mice were randomly selected from the control and model groups. The hippocampal tissue was removed and preserved in liquid nitrogen. If the sample quality inspection results were qualified, downstream experiments could be conducted.

Total RNA was extracted directly from cells or tissues using Trizol reagent and RNA was released after cell lysis. RNA and DNA were separated under acidic conditions. After the addition of chloroform, the sample was divided into aqueous and organic layers, with RNA present in the aqueous layer. After collecting the upper aqueous sample layer, the RNA was reduced by isopropanol precipitation.

The optical density (OD) and purity of the RNA were measured using a NanoDrop spectrophotometer. Few samples were subjected to agarose gel electrophoresis to determine whether the RNA was degraded.

The RNA samples were selected and sequenced using HiSeq2500 (American Illumina platform) to build libraries of mRNAs (mRNA library) and miRNAs (microRNA library). Comprehensive sequencing was performed after fragment removal, chain synthesis, end repair, A-tail and adapter connection, PCR amplification, cluster generation, and quality control.

### 2.8 Identification and analysis of differentially expressed miRNA and its target genes

Analysis of differentially expressed genes is an important part of comparative transcriptome sequencing, as well as the basis for excavating important regulatory miRNAs and searching for key genes to explain their biological molecular regulatory mechanisms. We analyzed the differentially expressed miRNAs and mRNAs in the chronic alcohol exposure and control groups. Furthermore, we predicted and annotated the target genes of differentially expressed miRNAs using bioinformatic methods to analyze the expression patterns of miRNAs and target genes and conducted a joint analysis with mRNA sequencing data to provide more information for exploring the molecular mechanism of miRNAs regulating chronic alcohol exposure.

We used Fragments Per Kilobase Million (FPKM) to calculate the abundance of known genes in different samples. For mRNA, |log2 fold change| ≥1 and *p* < 0.05 can be considered as differentially expressed. It was visualized by volcano and cluster diagrams.

### 2.9 miRNA-mRNA regulatory network construction

Target genes with differential expression of miRNAs were predicted using the TargetScan, miRTarBase, and miRDB databases. The target genes were subsequently intersected to construct a differentially expressed miRNA-mRNA regulation correlation network, and the regulatory network was visualized using Cytoscape_v3.9.0.

### 2.10 Gene ontology/Kyoto encyclopedia of genes and genomes (KEGG) enrichment analysis of targeting mRNAs

Gene ontology (GO) and KEGG enrichment analyses are the two most widely used gene function analysis strategies. GO can annotate and cluster gene functions from three levels: molecular function (MF), cell composition, and biological processes (BP), while KEGG collects a large amount of gene pathway information, which contains information such as gene metabolic pathways, products, and enzymes, which can help researchers explore the specific functions of genes from a deeper level. The basic unit of GO is a term corresponding to an attribute, whereas the basic unit of KEGG is a pathway.

### 2.11 Protein-protein interaction network construction

The STRING database covers a large amount of protein information, including protein interactions, three-dimensional structures, and related functional enrichment results. Multiple differentially expressed genes can be built using this database. A protein interacting with N proteins can assume a degree of “n.” The higher the degree score is, the more likely the protein is at a key node in the network structure. In this study, the top 30 key genes in degree rankings are in the Table 3.

### 2.12 qPCR verifies the expression of key mRNAs

Each group was randomly selected from the hippocampus of eight mice, and total RNA was extracted from the hippocampus. First, the RNA OD value (A260/A280) was detected with the ultra-trace nucleic acid protein analyzer (scandrop100), and then the Aidlab reverse transcription kit (TUREscript 1st Stand cDNA SYNTHESIS Kit) was used for reverse transcription operation using a 20 μL reaction system comprising 1,000 ng of total RNA, 4 μL of 5×RT reaction mix, 1 μL of Rondam primer/oligodT, 1 μL of TUREscript H- RTase/RI mix, and the reaction mix was raised to 20 μL with RNase Free dH2O. The reverse transcription reaction conditions used were as follows: after 40 min at 42°C and 10 min at 65°C, the cDNA was obtained and stored at −80°C. The acquisition of Ct value data in the reaction was set by a corrected threshold. The real-time qPCR method used actin as the internal reference gene, and the 2^-△△Ct^ method was used for relative quantification. The amplification conditions were as follows: 95°C for 3 min, 95°C for 10 s, and 60°C for 30 s, with 40 cycles of the reaction and primers for key mRNA (Table 1).

**TABLE 1:** Forward and reverse primer sequences of key mRNAs (5′to 3′).

Primer name	Forward primer sequence	Reverse primer sequence
Actin	GGC​TGT​ATT​CCC​CTC​CAT​CG	CCA​GTT​GGT​AAC​AAT​GCC​ATG​T
FOS	CAGAGCATCGGCAGAAGG	CCG​CTT​GGA​GTG​TAT​CTG​TC
EGR1	AGG​AGA​TGA​TGC​TGC​TGA​G	GTG​CTG​CTG​CTG​CTA​TTA​C

### 2.13 Statistical processing

The ClusterProfiler (v4.0.3) package of the R language was used to analyze the target genes in the miRNA-mRNA regulatory network for GO and KEGG pathway enrichment to search for terms or pathways with significantly enriched differential genes. When a term or pathway had a *p*-value < 0.05, the entry was considered significantly enriched. GraphPad Prism 8.0.2 was used to visualize the qPCR results. The *t*-test was used for inter-group comparison, and *p* < 0.05 was deemed statistically significant.

## 3 Results

### 3.1 Assessment of mouse learning and memory capabilities in each group

**FIGURE 1:**
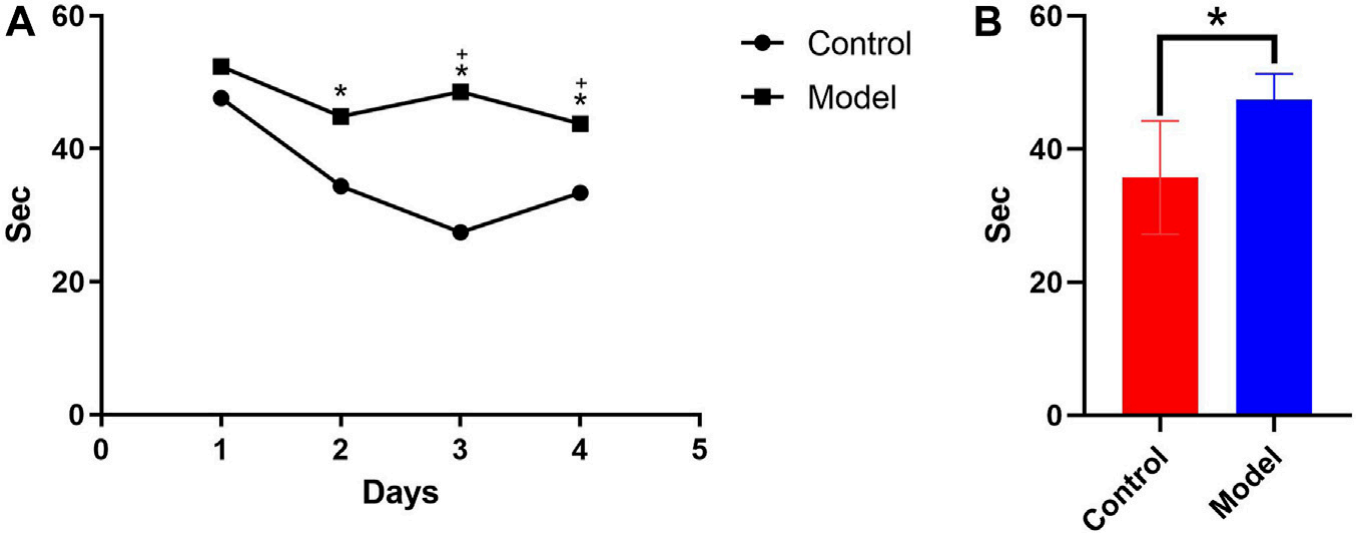
**(A)** Comparison of the 4-day incubation period of control and chronic alcoholic mice (model group). **(B)** Comparison of the mean latency of control and chronic alcoholic mice. (*p* < 0.05).

According to the water maze experiment results (Figure 1), there was no substantial difference in latency between the mice in each group on the first day (*p* > 0.05); however, from the second day onwards, the latency of the mice in the model group was significantly higher than that of the control group (*p* < 0.05), indicating that chronic alcohol exposure seemed to have some negative effects on their learning capacity. The latency of the mice in each group decreased as the number of empirical days increased (Figure 1A). The 4-day place navigation experiment data were analyzed and processed, and the results revealed that the model group had considerably higher latency than the control group (*p* < 0.05), indicating that the mice chronically exposed to alcohol had impaired learning functions. This indirectly demonstrated the successful initial construction of the mice model with alcohol addiction (Figure 1B).

### 3.2 Measuring the activity of the mitochondrial ATPase

**FIGURE 2:**
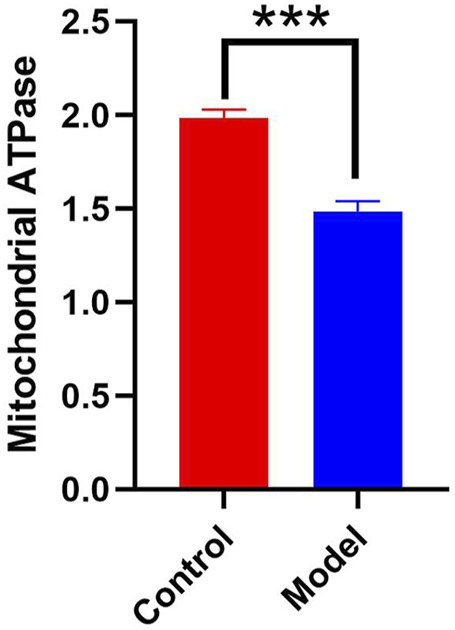
The activity of mitochondrial ATPase in hippocampal tissue (*p* < 0.01).

According to Figure 2, the hippocampal tissues of the mice in the model group had a significantly lower mitochondrial ATPase activity than that of the control group (*p* < 0.01). This finding suggests that chronic alcohol exposure impairs the ability of mitochondria to normally convert energy from food.

### 3.3 Determination of H_2_S content in the hippocampus of various groups of mice

As shown in Table 2, mice in the model group had significantly higher levels of H_2_S in their hippocampus than mice in the control group (*p* < 0.01). This finding suggests that chronic alcohol exposure can activate CBS, which catalyzes the production of significant amounts of H_2_S.

**TABLE 2:** H_2_S content in hippocampal tissue from mice in each group.

Group	N	H₂S content in the hippocampus (nmol/g)	t
C	8	35.47 ± 2.48	17.264
M	8	57.44 ± 3.04	

Remarks: Model (M) group compared to control (C) group, *p* < 0.01.

### 3.4 Identification of differentially expressed of miRNAs and mRNAs

**FIGURE 3:**
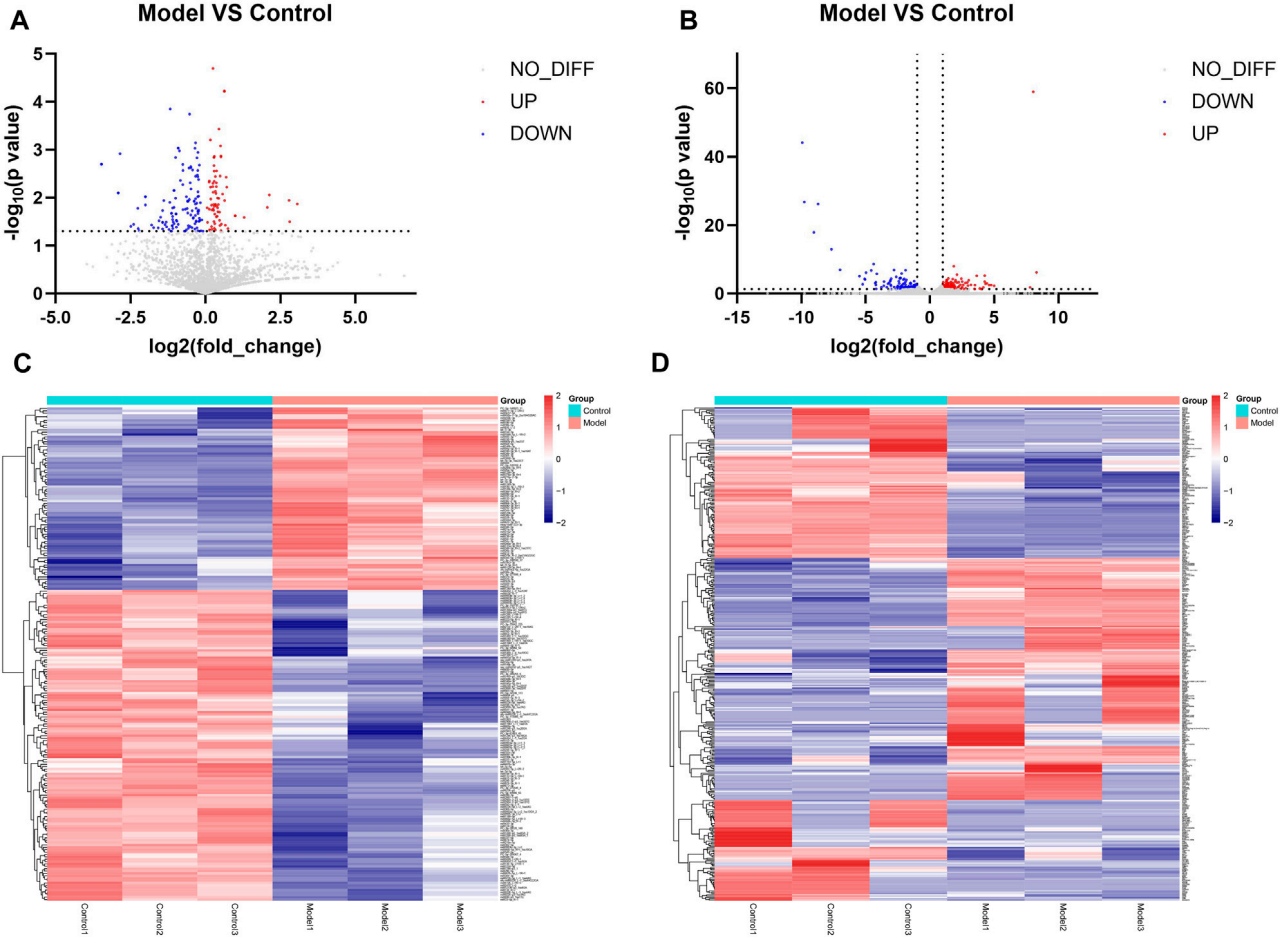
Results of identification of differentially expressed miRNAs and mRNAs **(A)** Volcano plot of differentially expressed miRNAs. Remarks: Red is upregulated miRNA, blue is downregulated miRNA, and gray is genes that do not have statistically significant differences. **(B)** Volcano plot of differentially expressed mRNA. Remarks: Red is upregulated miRNA, blue is downregulated miRNA, and gray is genes that do not have statistically significant differences. **(C)** Heatmap of differentially expressed miRNA. Remarks: Red is upregulated miRNA, blue is downregulated miRNA, darker color means higher degree of up/downregulation. **(D)** Heatmap of differentially expressed mRNA. Red is upregulated miRNA, blue is downregulated miRNA and darker color means higher degrees of up/downregulation.

Differential expression analysis was performed on the miRNA sequencing data with the screening conditions of *p* < 0.05. In total, 208 miRNAs were differentially expressed, of which 77 were upregulated and 131 were downregulated. Figures 3A, C show volcano plots and heatmaps of the differentially expressed miRNAs. The mRNA sequencing data were subjected to differential expression analysis, with the screening condition of a |log Fold change| ≥1 and *p* < 0.05. A total of 377 differentially expressed mRNAs were identified, of which 185 were upregulated and 192 were downregulated, and the volcano plot and heatmap of differentially expressed mRNAs are shown in Figures 3B, D.

### 3.5 Construction of miRNA-mRNA regulatory network

**FIGURE 4:**
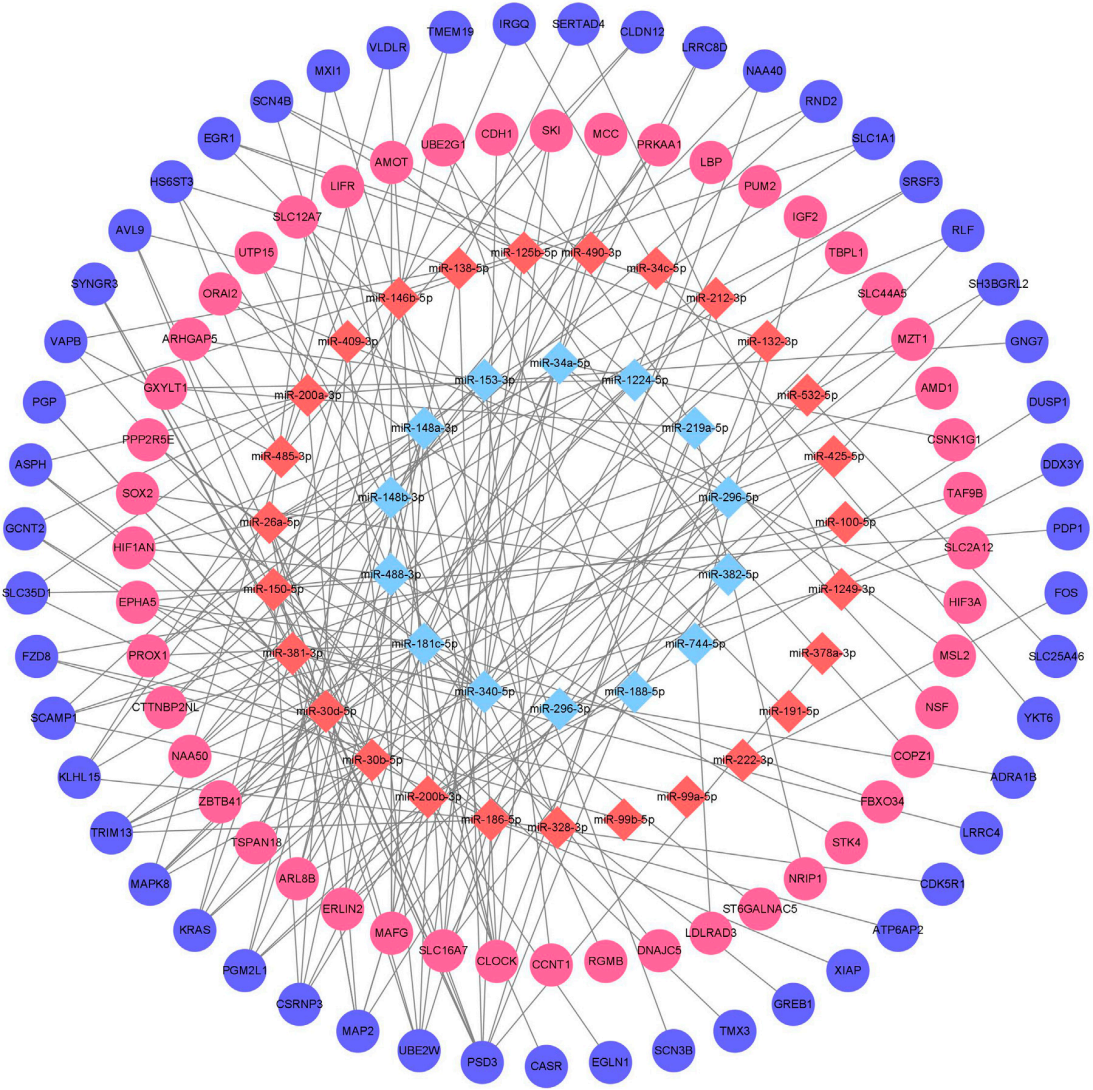
Chronic alcohol exposure miRNA-mRNA regulatory network Remarks: Red represents upregulation, blue represents downregulation, miRNA is represented by diamond-shaped sites and mRNA is represented by circular sites.

Downstream target prediction of differentially expressed miRNAs was performed using the Targetscan, miRTarBase, and miRDB databases, and 437 miRNA-mRNA interactions were detected by intersecting the results of the three databases. The target mRNAs were then intersected with the previously mentioned 377 differentially expressed mRNAs. The final miRNA-mRNA network contained 41 differentially expressed miRNAs and 99 differentially expressed mRNAs (Table 3). Cytoscape was used to build and show the miRNA-mRNA regulatory network (Figure 4).

**TABLE 3:** Partial miRNA-mRNA relationship pairs.

miRNA	Up/Down	mRNA	Regulation	Score
*miR-219a-5p*	Down	*GXYLT1*	Up	100
*miR-222-3p*	Up	*FOS*	Down	98
*miR-381-3p*	Up	*PSD3*	Down	96
*miR-153-3p*	Down	*SLC16A7*	Up	95
*miR-219a-5p*	Down	*CDH1*	Up	94
*miR-200b-3p*	Up	*PGM2L1*	Down	93
*miR-30b-5p*	Up	*TRIM13*	Down	92
*miR-532-5p*	Up	*KRAS*	Down	90
*miR-30b-5p*	Up	*SYNGR3*	Down	89
*miR-30b-5p*	Up	*PGP*	Down	87
*miR‐30b‐5p*	Up	*AVL9*	Down	86
*miR-340-5p*	Down	*RGMB*	Up	85
*miR-340-5p*	Down	*ZBTB41*	Up	85
*miR-148a-3p*	Down	*PRKAA1*	Up	82
*miR-425-5p*	Up	*SCAMP1*	Down	81
*miR-148a-3p*	Down	*CTTNBP2NL*	Up	78
*miR-148b-3p*	Down	*SLC12A7*	Up	75
*miR-212-3p*	Up	*EGR1*	Down	73
*miR-132-3p*	Up	*EGR1*	Down	73
*miR-188-5p*	Down	*UBE2G1*	Up	71
*miR-485-3p*	Up	*CLDN12*	Down	69
*miR-340-5p*	Down	*SOX2*	Up	68
*miR-488-3p*	Down	*ERLIN2*	Up	65
*miR-153-3p*	Down	*PROX1*	Up	63
*miR-191-5p*	Up	*EGR1*	Down	62
*miR-138-5p*	Up	*VAPB*	Down	60
*miR-186-5p*	Up	*NAA40*	Down	57
*miR-488-3p*	Down	*EPHA5*	Up	55
*miR-200b-3p*	Up	*DDX3Y*	Down	54
*miR-409-3p*	Up	*SCN4B*	Down	52

Score is the percentage of sites for this miRNA.

### 3.6 Enrichment analysis of mRNA target

**FIGURE 5:**
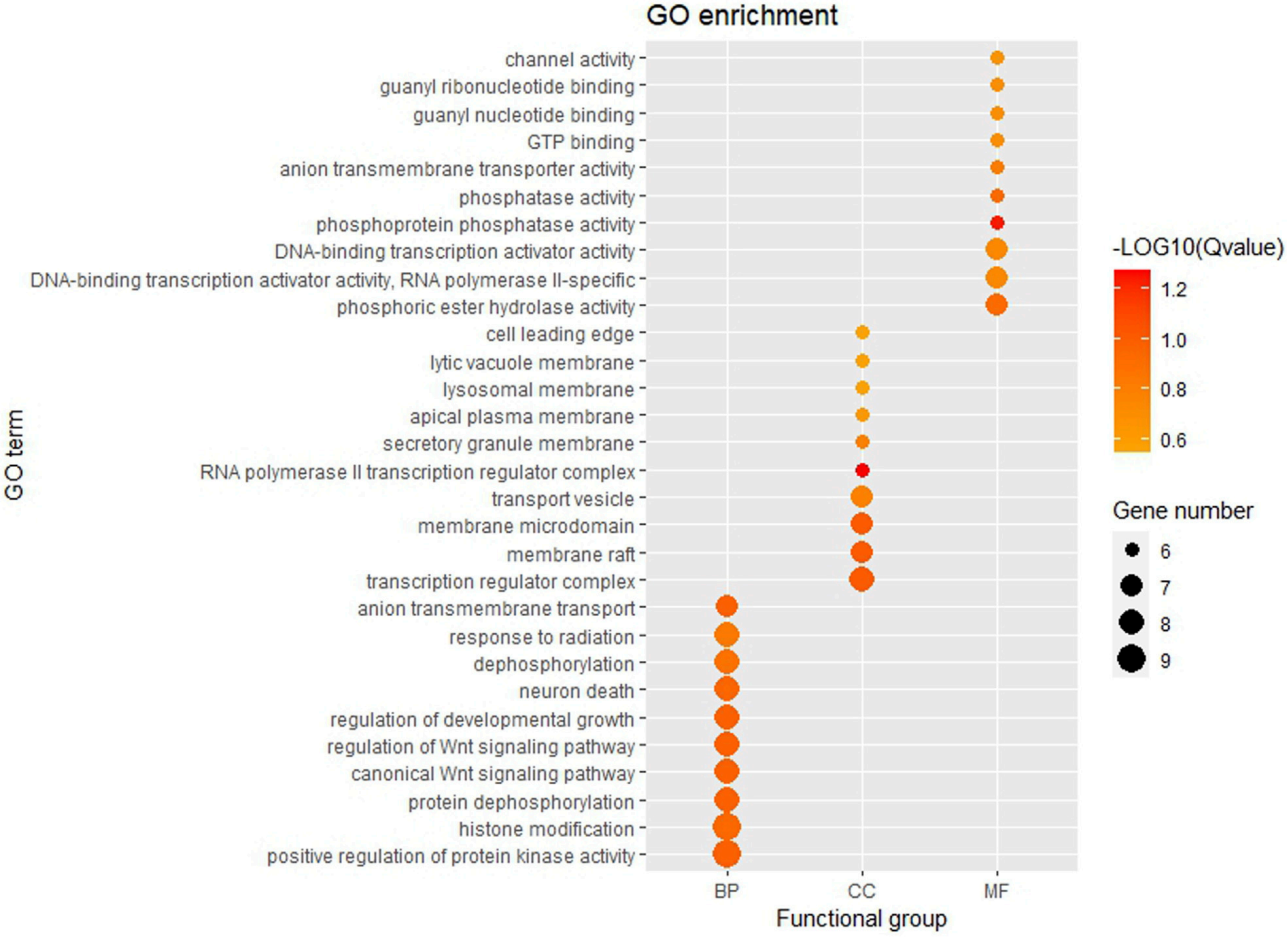
Bubble chart of GO enrichment Remarks: From left to right, there are BP, CC and MF. Larger dots represent more differential genes involved in the entry and darker colors represent more significant enrichment.

**FIGURE 6:**
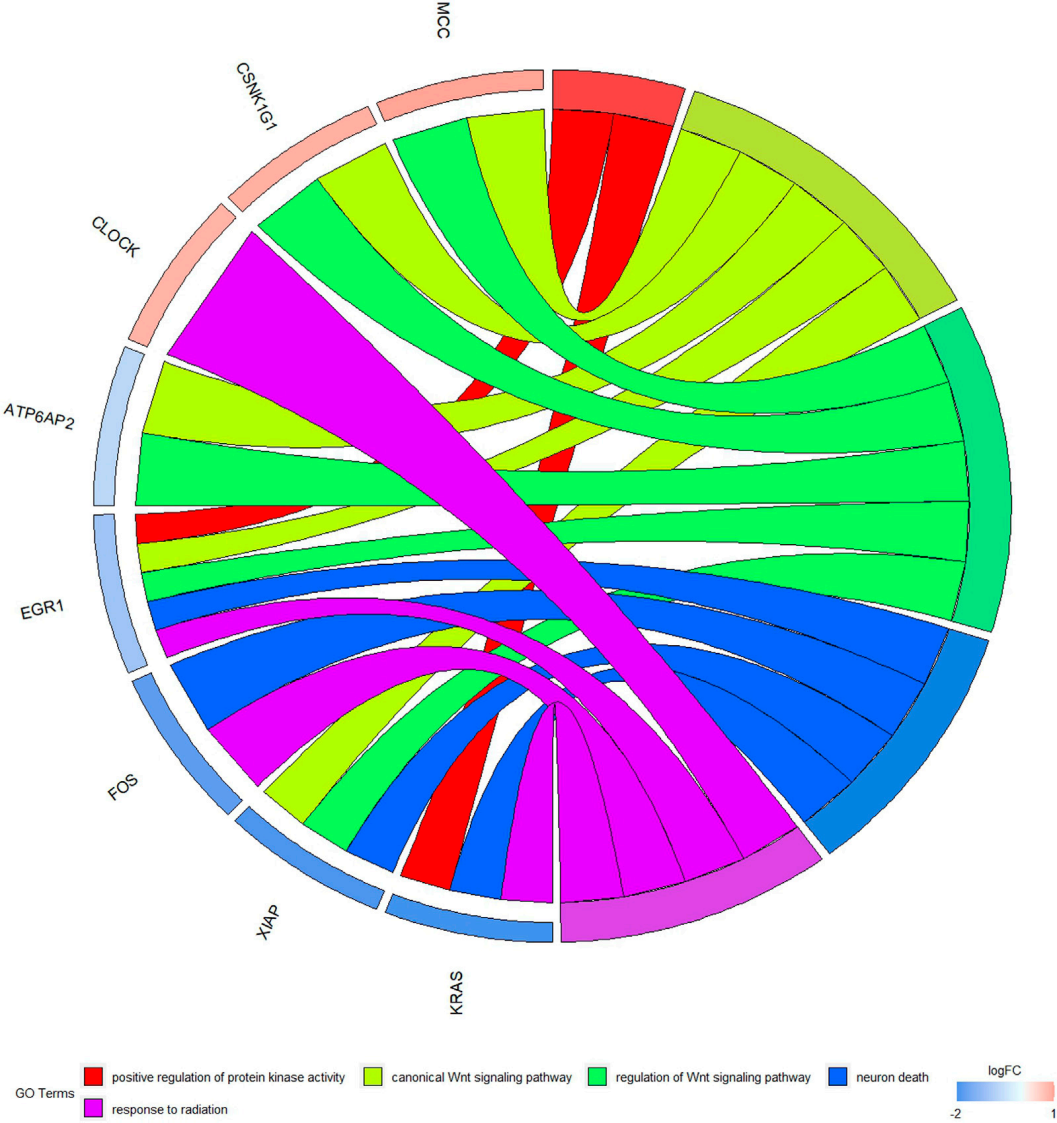
Chord diagram of GO enrichment Remarks: The chord diagram shows the top five most significantly enriched GO results.

**FIGURE 7:**
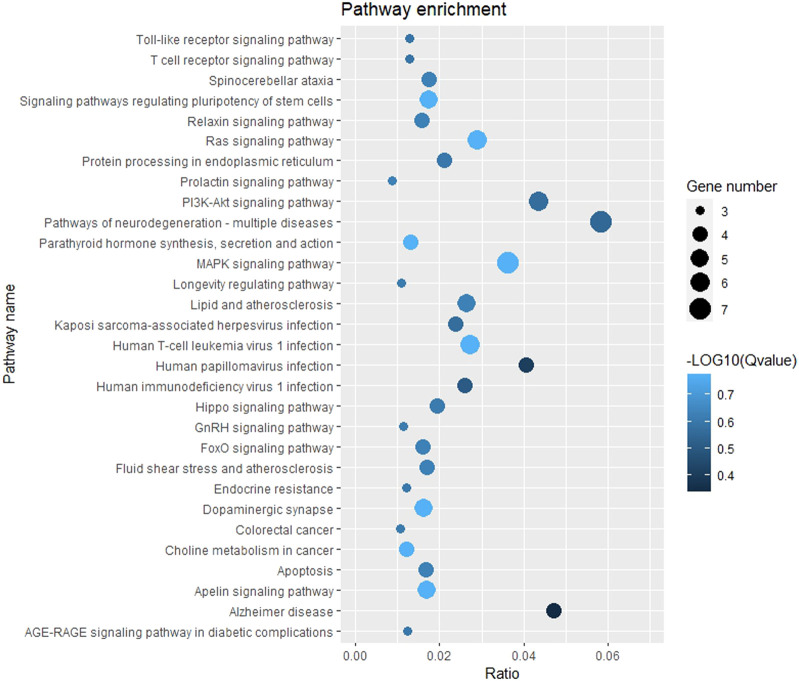
Bubble chart of KEGG pathway enrichment. Remarks: The larger dot means more differential genes are involved in the entry, and the darker the color means more significant enrichment.

The GO function and KEGG pathway enrichment analyses of target genes in the miRNA-mRNA regulatory network were performed using the cluster Profiler package in the R language. Approximately, 2,762 GO enrichment terms were obtained, including 2,190 BP terms, 248 cellular component (CC) terms, and 324 MF terms. BPs mainly comprised neuronal death, regulation of the Wnt signaling pathway, regulation of developmental growth, anion transmembrane transporter activity, and histone modification protein dephosphorylation; CC terms were mainly related to RNA polymerase II transcription regulator complex, transcription regulatory complex, and lysosomal membrane; and MFs comprised phosphoric ester hydrolase activity, DNA binding transcription activator activity, and phosphatase activity. The bubble chart displays the top ten enriched BP, CC, and MF terms (Figure 5). A chord diagram was constructed using the GO plot package in R to illustrate the top five findings, with the most significant enrichment of GO term functions to demonstrate a smaller selection of high-dimensional data (Figure 6). The KEGG pathway enrichment analysis yielded 190 signaling pathways, namely, the mitogen-activated protein kinase (MAPK) signaling pathway, pathways of neurodegeneration-multiple disease, phosphoinositide 3-kinase-protein kinase B (PI3K-Akt) signaling pathway, mitogen-activated protein kinase (Ras) signaling pathway, and dopaminergic synaptic pathway (Figure 7).

### 3.7 Construction of protein-protein interactions (PPI) network

**FIGURE 8:**
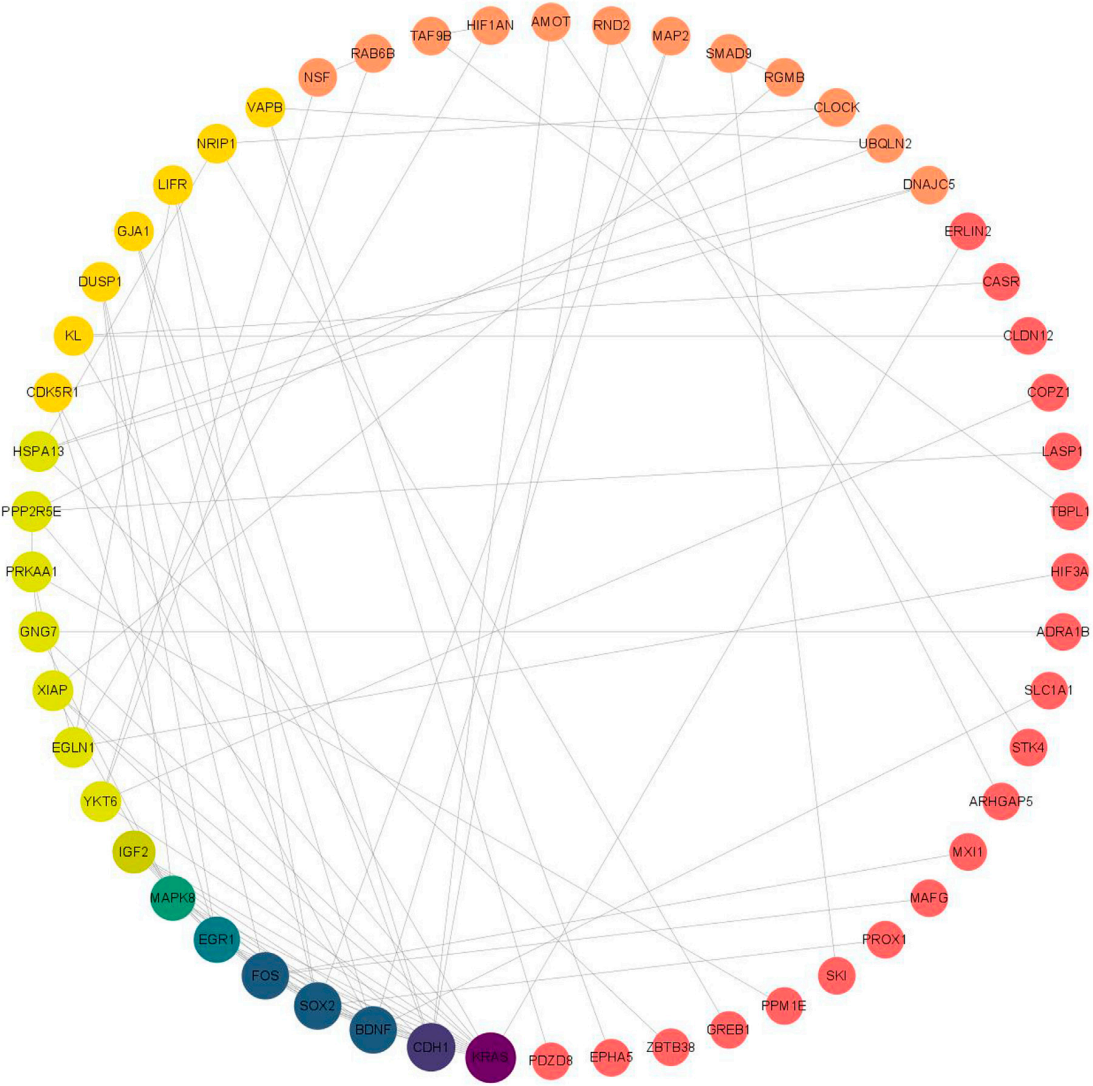
PPI network of target genes in miRNA-mRNA regulatory network.

The PPI network was analyzed using the STRING database for 118 target genes in the miRNA-mRNA regulatory network (Figure 8), and the PPI protein interaction network constructed in this study consisted of 117 nodes and 96 edges (Table 4 shows the mRNAs in the top 30 of degree). Then, we delete the scattered nodes, leaving the central part of the network. Among them, the Kirsten rat sarcoma viral oncogene homolog (KRAS), cadherin 1 (CDH1), Fos proto-oncogene, transcription factor activator protein-1 subunit (FOS), SRY-box transcription factor 2 (SOX2), brain-derived neurotrophic factor (BDNF), and early growth response 1 (EGR1) were the top six nodes with the highest degree scores, and thus were the key node genes in this PPI network.

**TABLE 4:** Top 30 mRNAs of degree value.

mRNA	Degree	mRNA	Degree	mRNA	Degree
*KRAS*	13.0	*HSPA13*	4.0	*CDK5R1*	3.0
*CDH1*	11.0	*YKT6*	4.0	*GJA1*	3.0
*SOX2*	10.0	*PPP2R5E*	4.0	*SMAD9*	2.0
*FOS*	10.0	*XIAP*	4.0	*RGMB*	2.0
*BDNF*	10.0	*GNG7*	4.0	*RAB6B*	2.0
*EGR1*	9.0	*VAPB*	3.0	*NSF*	2.0
*MAPK8*	8.0	*DUSP1*	3.0	*UBQLN2*	2.0
*IGF2*	6.0	*NRIP1*	3.0	*TAF9B*	2.0
*PRKAA1*	4.0	*LIFR*	3.0	*HIF1AN*	2.0
*EGLN1*	4.0	*KL*	3.0	*CLOCK*	2.0

The higher the degree of mRNAs, the more crucial the role they play in chronic alcoholism.

### 3.8 Detection of the relative expression of key mRNAs by qPCR

**FIGURE 9:**
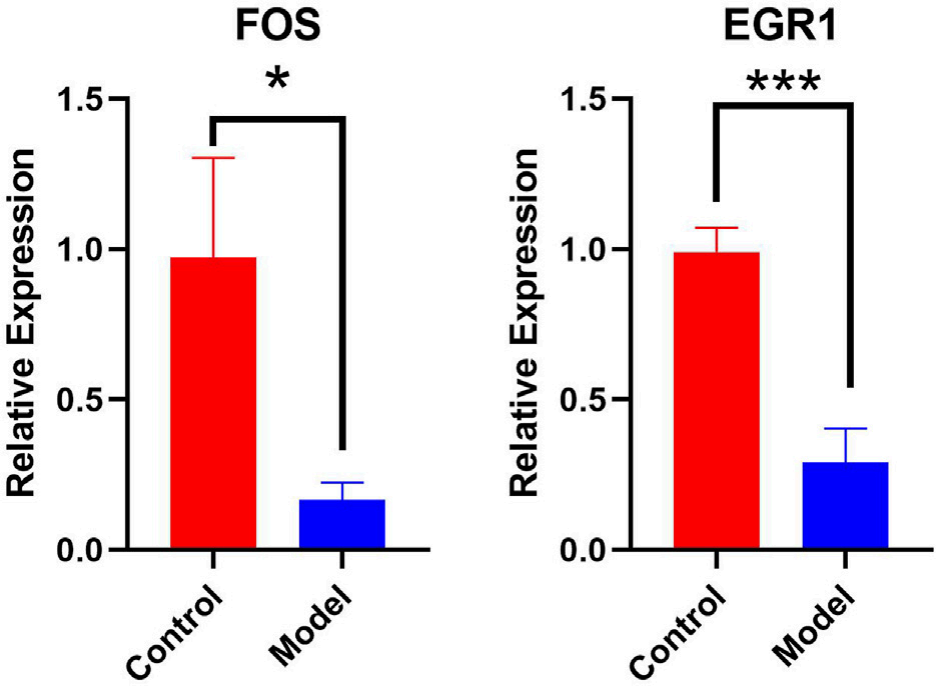
Relative expression of key genes FOS, EGR1 mRNA.

Real-time qPCR was used to detect key genes, such as *FOS* and *EGR1*. The results indicated that *FOS* and *EGR1* mRNA expression in the hippocampus of chronic alcoholic mice was significantly decreased (*p* < 0.05), suggesting that chronic alcohol exposure alters the expression of several important mRNAs in the hippocampus of mice. Furthermore, GraphPad Prism software was used to display the qPCR results (Control refers to control mice, Model refers to mice in the chronic alcohol exposure group). Visualization results are shown in Figure 9).

## 4 Discussion

The adverse effects of chronic alcoholism on neurodegenerative diseases are widespread worldwide and increase annually. Epidemiological investigations have shown that alcohol consumption is an important risk factor for neurodegeneration (Hammoud and Jimenez-Shahed, 2019). In a previous study, we confirmed that alcohol exposure causes neurodegeneration in rats (Du et al., 2014). In this study, the water maze experiment showed that the learning ability of mice chronically exposed to alcohol was impaired. Simultaneously, the activity of mitochondrial ATPase in mice was detected, and the results showed that the activity of ATPase in mice with chronic alcohol exposure was significantly decreased, suggesting that alcohol exposure may cause nerve cell death through mitochondrial damage. Studies have shown that chronic alcohol exposure mediates calcium overload through the N-methyl-d-aspartic acid receptor, causing mitochondrial damage (Du et al., 2019) and inducing autophagy (Maiese, 2020). Alcohol consumption may lead to neurodegeneration through mitochondrial autophagy. In this study, H_2_S was detected in the hippocampus, suggesting that chronic alcohol exposure can activate CBS and catalyze the formation of large amounts of H_2_S. Our previous study showed that H_2_S can cause calcium overload, leading to mitochondrial damage and ultimately neurodegeneration (Du et al., 2018). In this study, through the analysis of miRNA expression profiles in mice and further combined with the mRNA target gene information regulated by miRNAs, a miRNA-mRNA regulatory network related to chronic alcohol exposure in mice was constructed, and the potential genes involved in mice with chronic alcohol exposure were discussed. Through the identification and classification of miRNAs, 208 differentially expressed miRNAs were identified, including 77 upregulated differentially expressed miRNAs and 131 downregulated miRNAs. These miRNAs may play a key role in chronic alcohol exposure in mice.

The GO enrichment analysis results showed that the potential target genes of miRNAs differentially expressed in mice with chronic alcohol exposure were mainly related to the regulation of the Wnt signaling pathway, neuronal death, and DNA-binding transcription activator activity (Figure 5). The results of functional enrichment showed that the potential target genes of miRNAs were mainly related to the MAPK signaling pathway, PI3K-Akt signaling pathway, pathways of neurodegeneration-multiple disease, etc. (Figure 7). Studies have shown that the Wnt signaling pathway, phosphatidylinositol 3-kinase (PI3K), and protein kinase B (Akt) are related to autophagy, indicating that differentially expressed miRNAs in mice chronically exposed to alcohol may cause neurodegeneration by participating in autophagy (Maiese, 2017). The PPI network analysis of the target genes showed that the top genes with the highest degrees were *KRAS*, *CDH1*, *FOS*, *SOX2*, *BDNF*, and *EGR1*, which were highly significant in the PPI network, suggesting that chronic alcohol exposure was closely related to these six genes (Figure 8). Several studies have shown that *KRAS* and other genes have different effects on nerves. For example, Kiyota et al. showed that KRAS induced robust retinal ganglion cells axon regeneration (Kiyota et al., 2023).

Real-time qPCR results showed that *FOS* and *EGR1* mRNA expression levels were significantly decreased in the mice with chronic alcohol exposure, and the differences were statistically significant (*p* < 0.05 or *p* < 0.01). This suggests that chronic alcohol consumption affects the expression of key mRNAs. Therefore, *FOS* and *EGR1* play important roles in chronic alcohol exposure. The *FOS* gene family consists of four members: *FOS*, *FBJ* murine osteosarcoma viral oncogene homolog B (*FOSB*), FOS Like 1 (*FOSL1*), and FOS Like 2 (*FOSL2*). These genes encode leucine zipper proteins that can dimerize with proteins of the JUN family, thereby forming the transcription factor complex, activator protein-1 (AP-1) (Renaud et al., 2014). The FOS proteins are implicated in the regulation of cell proliferation, differentiation, and transformation. Furthermore, *c-FOS* is a member of the *FOS* gene family and plays an important role in the regulation of cell growth, proliferation, division, differentiation, and death. Studies have shown that increased expression of the synapse-associated protein *c-FOS* in the hippocampus is associated with memory function. The down-regulation of FOS expression in this study, to some extent, explains the mechanism of alcohol exposure damage to learning and memory ability (Acquarone et al., 2024). Similarly, the down-regulation of EGR1 may also play a role in neurodegenerative changes. Research indicates that in Alzheimer’s disease model mice, EGR1 is downregulated when the mice display cognitive deficits (Koldamova et al., 2013).

Additionally, *FOS* and *EGR1* are regulated by H_2_S and calcium ions; therefore, it is speculated that they are correlated with neurodegeneration caused by chronic alcohol exposure. Hydrogen sulphide is a gaseous neurotransmitter discovered in recent years that has many important physiological functions, such as neuroregulation, vasoconstriction regulation, inflammatory response regulation, and cell growth. Li et al. (2021) found that H_2_S donors could inhibit the activity of the nuclear factor kappa-B pathway and ultimately reduced the expression of *c-FOS* in the brain. Han et al. (2005) found that when NaHS (an H_2_S donor) was used in high-fever convulsion (FS) rats, the concentration of H_2_S in the hippocampus increased, *FOS* expression level decreased, and neuronal excitability decreased. Alcohol exposure may contribute to the generation of H_2_S in the hippocampus, and lead to the down-regulation of c-FOS and the decrease of neuronal excitability, and further lead to learning and memory damage.

Other studies have demonstrated that the key miRNAs identified in this study (*miR-222-3p, miR-132-3p*, and *miR-212-3p*) are involved in autophagy, neurodegeneration, neuroinflammation, ferroptosis and other processes. Therefore, the miRNAs identified in this study may be related to processes caused by alcohol exposure. There is evidence that *miR-222-3p* is significantly up-regulated in the plasma of Huntington patients, suggesting that *miR-222-3p* is related to neurodegeneration and is a promising therapeutic target (Díez-Planelles et al., 2016). Additionally, early neurogenesis is the process by which neurons are produced from stem progenitor cells, and neurogenesis can promote the repair of the nervous system. Zhai et al. (2020) demonstrated that upregulation of *miR-212-3p* could result in the slowdown of early neurogenesis by disrupting AKT/mammalian target of rapamycin (mTOR) pathway activation, while our research shows that *miR-212-3p* was upregulated in chronically alcoholic mice, which may be another way about chronic alcoholism leads to nerve damage (Zhai et al., 2020). Inflammation is a common component of acute CNS injuries, such as ischemia and degenerative diseases, such as Alzheimer’s disease (Hutchison et al., 2013). Glial cells play an important role in the inflammatory reactions in the CNS (Maiese, 2023). Previous studies have shown that *miR-132-3p* has toxic effects on neurons by targeting glutaredoxin, enhancing the activation of microglia and promoting the release of inflammatory cytokines, leading to neuroinflammation (Gong et al., 2022).

Based on the above results and the literature, the current studies indicate that *FOS* is independently regulated by *miR-222-3p* (Table 3). Additionally, *EGR1* was regulated by three miRNAs (*miR-132-3p, miR-212-3p,* and *miR-191-5p*), and the results of the KEGG pathway enrichment analysis showed that *FOS* and *EGR1* were related to neurodegeneration. It is speculated that *miR-222-3p* may be involved in the regulation of neurodegeneration by regulating *FOS*, and that the regulation of *EGR1* by the three miRNAs is also involved in this regulation. This study has several findings that support this idea. Leinders et al. (2016) found that changes in *miR-132-3p* expression can alter neuronal-glial interactions and neuronal function, which may lead to neuropathic pain, possibly because miR-132-3p promotes neuronal damage by downregulating *EGR1* expression in nerve cells. Both *miR-132* and *miR-212* are homologous microRNAs (Aten et al., 2019). Studies have shown that *miR-132-3p/212-3p* play an important role in regulating neuronal morphogenesis and promoting dendrite maturation, and are negatively correlated with the expression of neuronal apoptosis-related genes, such as Bcl-2 interacting mediator of cell death (BIM).

Analysis of the miRNA-mRNA interaction network enriched our understanding of the negative regulation mode and targeting specificity of miRNAs in chronic alcoholic mice and provided information for further understanding of the regulatory function of miRNAs in chronic alcoholic mice. Additionally, four miRNAs associated with neuronal death in chronic alcoholic mice were detected in this study, among which *miR-191-5p* was newly discovered in this study, whereas *miR-222-3p, miR-132-3p*, and *miR-212-3p* were discovered in previous studies (Huang et al., 2018; Qazi et al., 2021). The miRNAs involved in the regulation of these mRNAs were identified through interaction network analysis (Figure 8) and deserve further study.

Further studies are required to determine the relationship between key miRNAs and chronic alcohol exposure. However, this study has some limitations. First, the sample size was relatively small; however, we used random sampling and multiple trials to minimize bias. Additionally, our findings represent a preliminary step toward revealing the miRNA-mRNA network in chronic alcohol exposure. Although the breadth of the analysis was sufficient, the depth was insufficient. Therefore, further animal experiments and clinical trials are required to confirm the clinical utility of these four core miRNAs and their target mRNAs as diagnostic markers of chronic alcoholism. The miRNA-mRNA network showed that only the miR-191-5p-EGR1 subnetwork was a newly discovered miRNA-mRNA network. Although our study achieved remarkable findings through a series of bioinformatic analyses, more laboratory experiments and large-scale clinical trials are required in the future. Moreover, the relationship between miR-212-3p-EGR1, miR-222-3p-FOS, and miR-132-3p-*EGR1* needs to be validated in large-scale studies of patients with chronic alcoholism to verify their diagnostic value and evaluate their potential clinical significance by comparing their clinical and pathological features. In the future, other subnetworks of miRNA-mRNA networks should be further explored and verified by *in vivo* and *in vitro* studies.

## 5 Conclusion

In this study, a model of chronic alcohol exposure was successfully established. The hippocampal tissues of mice with chronic alcohol exposure and control mice were sequenced using high-throughput sequencing technology, providing relevant upstream evidence for the pathogenesis of alcohol exposure. Many differentially expressed miRNAs and mRNAs were identified in the hippocampus of mice with chronic alcohol exposure by screening and performing comparisons using high-throughput sequencing technology. These results suggest that *miR-222-3p-FOS* and *miR-132-3p/miR-212-3p/miR-191-5p-EGR1* are likely to be involved in neurodegenerative processes and related to learning and memory impairment caused by alcohol exposure. However, further experiments are required to verify the relationships between these factors and their possible mechanisms of action.

## Data Availability

The original contributions presented in the study are included in the article/supplementary material, further inquiries can be directed to the corresponding author.
